# Supplementary material for: The Significance of Angling in Stress Reduction during the COVID-19 Pandemic—Environmental and Socio-Economic Implications
Source: Int J Environ Res Public Health. 2022 Apr 5;19(7):4346. doi: 10.3390/ijerph19074346 (PMC8998353; doi:10.3390/ijerph19074346)
Supplement: Supplementary file 1 [file ijerph-19-04346-s001.zip › ijerph-1644119-supplementary.pdf]

Table S1. The full survey questionnaire.

No.	Question	Options				
**I. Sociodemographic, economic, and engagement in angling**						
1	Age	Type in the correct value according to your best knowledge				
2	Income (per month)					
3	Domicile - place of residence (in thousands of inhabitants)					
4	Experience - how long have you been fishing for? (in years)					
5	Avidity - how often do you fish? (days in a year)					
6	Costs - how much money do you spend on your hobby (equipment expenses, licenses, travel, etc.) per year (in Euro)					
7	Distance - what is the distance you travel to your most visited fishing spot? (in kilometers)					
8	Education	Primary	Secondary	Higher		
9	Tourism - is fishing the main motive of your tourist trips?	Yes	No			
10	Association - are you a member of an angling organization/association?	Yes	No			
11	Urban site - is the fishing spot you most often visit in an urban area?	Yes	No			
**II. Perceptions and behaviors towards angling and life attitudes during the SARS-CoV-2 virus pandemic** (how much do you agree with the statement?)*						
1	Fishing reduces my stress about the pandemic	1	2	3	4	5
2	During the pandemic period I fished more often	1	2	3	4	5
3	I am concerned about getting sick (or getting sick again) from Covid-19	1	2	3	4	5
4	I have a positive attitude towards vaccination against Covid-19	1	2	3	4	5
5	My mental condition during the pandemic period has improved	1	2	3	4	5
6	My physical condition during the pandemic period has improved	1	2	3	4	5
7	I fish with my family	1	2	3	4	5
8	I have limited contact with my family during the pandemic period	1	2	3	4	5
9	I fish with my friends	1	2	3	4	5
10	I have limited contact with my friends during the pandemic period	1	2	3	4	5
11	I fish alone	1	2	3	4	5
12	I feel exposed to Covid-19 infection at the fishing spot	1	2	3	4	5
13	I have limited outdoor activities during the pandemic period	1	2	3	4	5

*If you do not agree with the statement, please circle "1" (Strongly disagree) or "2" (Disagree). If you agree with the statement, please circle "5" (Strongly agree) or "4" (Agree). If you do not have an opinion on a given topic or it is difficult to determine it, then please circle "3" (I have no opinion - neutral).

Table S2. Sociodemographic, economic, and engagement in the angling of the surveyed anglers with the number and percentage of respondents (N = 564).

Characteristic & structure of categories		N	%	MoE* (%)
**I. SOCIODEMOGRAPHIC AND ECONOMIC**				
1. Age (years)				
A_I	Less than 25	81	14.4	7.7
A_II	25-40	240	42.6	6.3
A_III	41-65	207	36.7	6.6
A_IV	66-85	36	6.4	8.0
2. Income (monthly)				
Inc_I	Less than 500€	129	22.9	7.3
Inc_II	500-1000€	291	51.6	5.7
Inc_III	1000-2000€	99	17.6	7.5
Inc_IV	More than 2000€	45	8.0	7.9
3. Domicile (in thousands of inhabitants)				
Dom_I	Village	138	24.5	7.2
Dom_II	Town to 5	45	8.0	7.9
Dom_III	A city 5 to 50	171	30.3	6.9
Dom_IV	A city 51-100	54	9.6	7.9
Dom_V	A city over 100	156	27.7	7.0
4. Education (level)				
Edu_I	Primary	93	16.5	7.5
Edu_II	Secondary	249	44.1	6.2
Edu_III	Higher	222	39.4	6.4
**II. ENGAGEMENT IN ANGLING**				
1. Experience (years)				
Exp_I	Less than 5	51	9.0	7.9
Exp_II	5-10	96	17.0	7.5
Exp_III	11-20	129	22.9	7.3
Exp_IV	21-30	147	26.1	7.1
Exp_V	31-40	69	12.2	7.7
Exp_VI	More than 40 years	72	12.8	7.7
2. Avidity (frequency of angling)				
Avi_I	A few times a year	54	9.6	7.9
Avi_II	A dozen or so times a year	72	12.8	7.7
Avi_III	About 2-3 times a month	126	22.3	7.3
Avi_IV	About once a week	174	30.9	6.9
Avi_V	A few times a week	138	24.5	7.2
3. Costs (per year)				
Cos_I	Up to 25€	36	6.4	8.0
Cos_II	25-100€	60	10.6	7.8

Cos_III	100-250€	171	30.3	6.9
Cos_IV	251-500€	150	26.6	7.1
Cos_V	500-1000€	72	12.8	7.7
Cos_VI	More than 1000€	75	13.3	7.7
4. Distance (to most often fished spot in km)				
Dis_I	Less than 5	153	27.1	7.0
Dis_II	6-10	102	18.1	7.5
Dis_III	11-30	171	30.3	6.9
Dis_IV	31-50	69	12.2	7.7
Dis_V	51-70	36	6.4	8.0
Dis_VI	More than 70	33	5.9	8.0
5. Fishing is the main tourism motive				
TOUR_Y	Yes	270	47.9	6.0
TOUR_N	No	294	52.1	5.7
6. Affiliation in angling association				
AFF_Y	Yes	459	81.4	3.6
AFF_N	No	105	18.6	7.4
7. Most visited angling spot is in the urban area				
URB_Y	Yes	147	26.1	7.1
URB_N	No	417	73.9	4.2

* Margin of sampling error (%) at 95% confidence interval
